# Cutaneous Metastases and Long-Term Survival of a Patient With Clear Cell Renal Carcinoma

**DOI:** 10.7759/cureus.23598

**Published:** 2022-03-29

**Authors:** Marta Vilaça, Fátima Braga, Alexandra Mesquita

**Affiliations:** 1 Oncology, Hospital Pedro Hispano, Matosinhos, PRT

**Keywords:** long-term survival, target therapy, predictors of response, cutaneous metastasis, metastatic renal cell carcinoma

## Abstract

The treatment landscape of metastatic renal cell carcinoma (mRCC) has changed in the last decade with improvements in overall survival. Overall survival ranges from 57 months in good-to-intermediate prognosis patients to 19 months in poor prognosis patients. The most frequent sites of metastasis are the lungs, bone, distant lymph nodes, liver, adrenal, and brain. Cutaneous metastases are rare and represent an end-stage disease with a worse prognosis. Studying long-term survivors of mRCC can help clinicians to identify potential predictors of response to targeted therapy and define the best treatment sequences in this setting. In this case, we report a 59-year-old man with a good mRCC prognosis who is alive 156 months after the diagnosis of mRCC, 108 months with cutaneous metastases. The patient underwent five treatment lines, with good tolerance and quality of life. This therapeutic sequence was based on new treatment options and new evidence concerning mRCC.

## Introduction

Renal cell carcinoma (RCC) is the 14th most common cancer worldwide, with more than 430,000 new cases and 179,000 deaths in 2020, with a notable increase in incidence over the past several years [1]. It is a heterogeneous group of histopathological entities, and clear cell RCC (ccRCC) is the most common, accounting for 70-75% of all renal tumors [2]. Surgical resection is the standard of care in localized diseases. Although it is potentially is curative, in about 20-40% of the patients, the disease will relapse over time. In 25-30% of patients, RCC is diagnosed in the advanced stage with distance metastasis [2,3]. The most frequent sites of metastases are the lungs, bone, distant lymph nodes, liver, adrenal, and brain [3]. Cutaneous metastases from RCC are rare, with an incidence of 3%, and usually occur in an advanced stage [4].

The prognosis of metastatic RCC (mRCC) depends greatly on risk factors and is changing based on new treatments and new combinations [5]. According to a real-life study, the overall survival of patients with good-to-intermediate prognosis is 57 months, and 19 months in patients with a poor prognosis due to new treatment modalities [6,7]. Sequential treatments with vascular endothelial growth factor tyrosine kinase inhibitors, inhibitors of the mammalian target of rapamycin pathway (imTOR), and, more recently, immunotherapy are recommended depending on patient prognosis, although the ideal sequence has not been determined [8]. The goal of therapy for patients with mRCC is to prolong survival while maintaining a good quality of life, which should be taken into account when choosing second-line and later-line treatments. In patients with a good prognosis, the treatment sequence is not well established. Identifying the best treatment sequence and potential biomarkers of response can contribute to a treatment decision [9,10].

This case reports the long-term survival of an mRCC patient with local recurrence, cutaneous, pulmonary, and lymphatic metastasis who is alive 159 months after metastatic disease diagnosis. The patient underwent five different sequential treatment lines, with good tolerance and quality of life.

## Case presentation

A 59-year-old man with an Eastern Cooperative Oncology Group (ECOG) performance status 0 and no relevant medical history presented in February 2006 with sudden macroscopic hematuria and right flank pain. The computed tomography (CT) scan showed a tumor measuring 150 mm in the great dimension of the superior right kidney without evidence of distance metastasis suggestive of RCC. An open right radical nephrectomy was performed, and pathological examination revealed a pT2N0M0 Fuhrman grade III, ccRCC, and the margins were clear of tumor after surgery. Following the surgery, he remained under active follow-up.

Two years later, the CT scan showed local recurrence, with a new lesion measuring 28 mm in great dimensions in the surgical site and metastasis to the left kidney. A biopsy revealed metastasis of RCC. Surgery was not an option because the lesions had invaded the inferior vena cava. The International Metastatic RCC Database Consortium (IMSDC) risk score was 0 (favorable risk), and the oncology multidisciplinary team decided to start systemic therapy. Therefore, the patient started first-line treatment with sunitinib, a tyrosine kinase inhibitor, 50 mg daily for four weeks, followed by two weeks off. Due to grade 2 acute kidney toxicity, hypertension, and hypothyroidism according to Common Terminology Criteria for Adverse Events version 5, sunitinib was reduced to 37.5 mg daily for four weeks on and two weeks off. He completed five years of this treatment with good tolerance and stable disease on a CT scan.

In 2011, due to disease progression with enlargement of the right metastatic nodule, he started second-line treatment with everolimus, an mTOR inhibitor, 10 mg/daily. Because he developed grade 2 hypertension and acute-on-chronic kidney disease, everolimus was reduced to 5 mg daily. He completed six years of this treatment with stable disease on CT scan and good tolerance.

**Figure 1:**
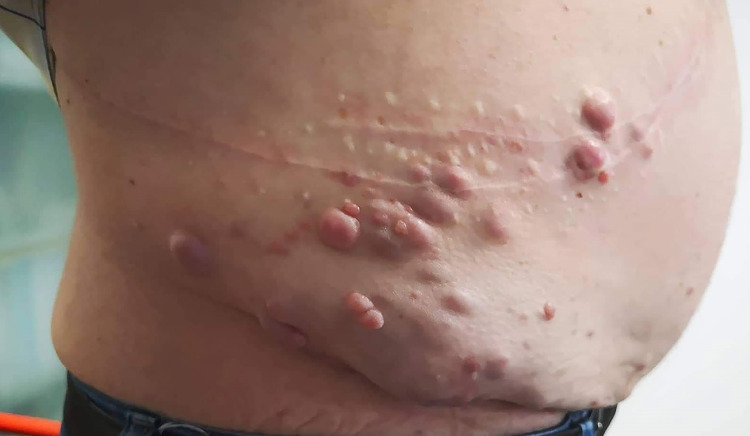
Cutaneous metastasis of ccRCC. ccRCC: clear cell renal cell carcinoma

**Figure 2:**
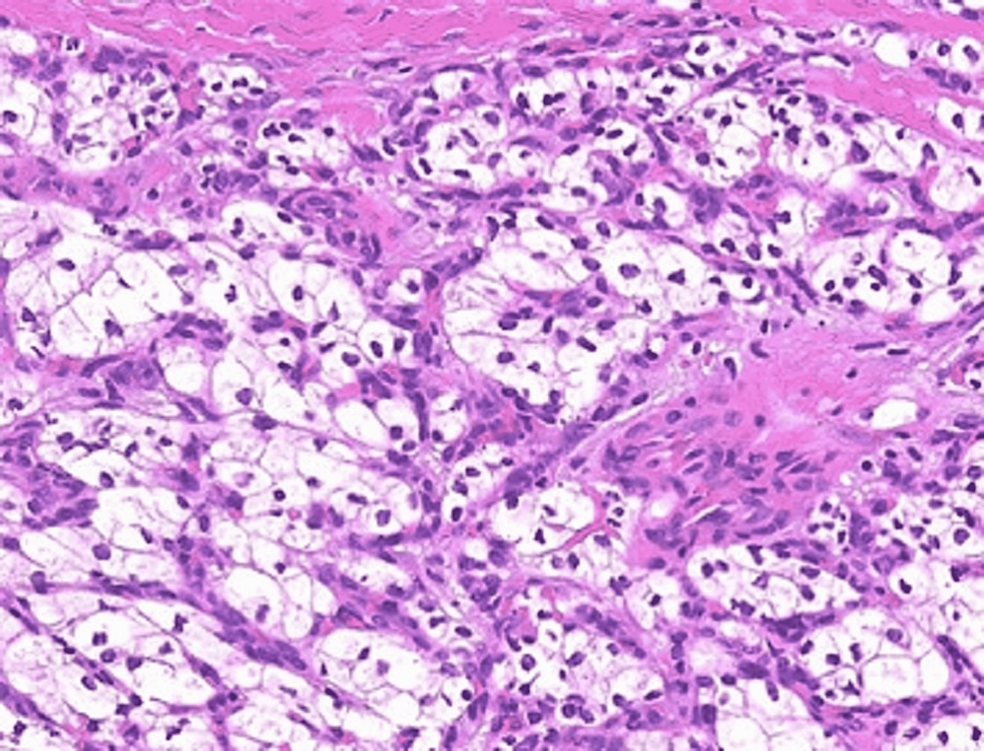
Histology of skin punch biopsies with large cells and clear cytoplasm, consistent with ccRCC (hematoxylin and eosin stain). ccRCC: clear cell renal cell carcinoma

**Figure 3:**
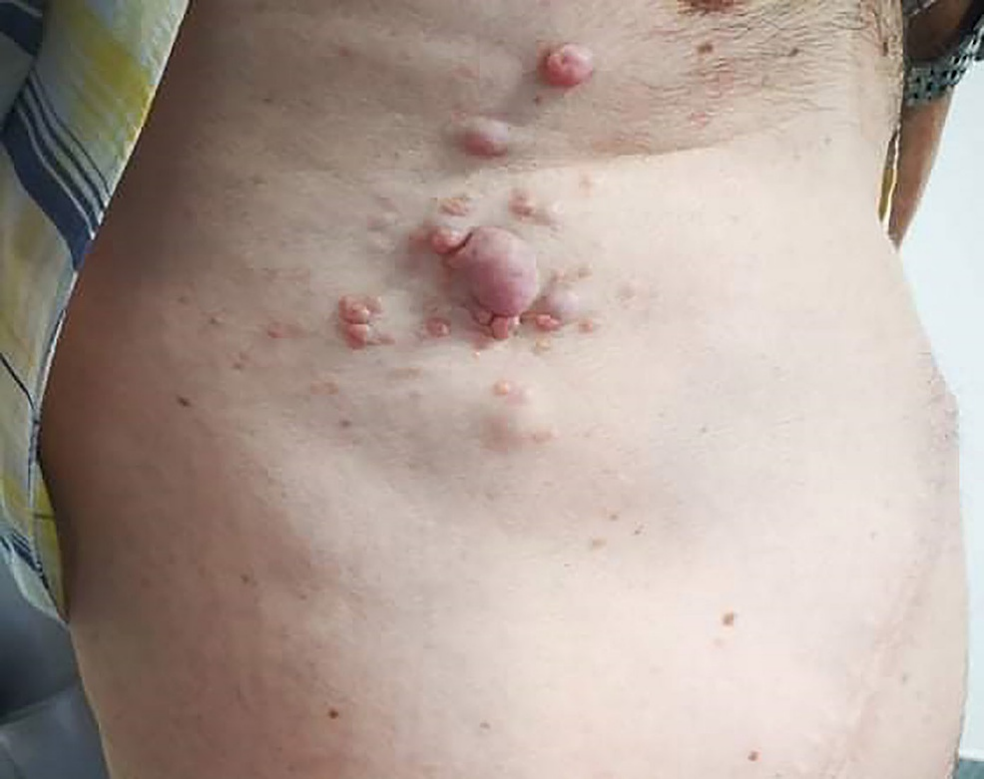
Cutaneous metastasis at the thoracic wall.

**Figure 4:**
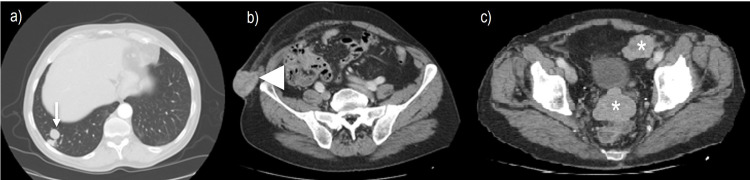
(a) Axial contrast-enhanced CT scan at the level of the thoracic outlet shows metastatic lesions (long arrow). (b) Axial contrast-enhanced CT scan at the pelvic level showing cutaneous metastasis. (c) Axial contrast-enhanced CT scan at the pelvic level showing solid nodules surrounding the spermatic cord (short arrow). CT: computed tomography

In 2017, the patient presented with pain and new violaceous skin lesions on the right flank near the surgical scar (Figure 1). A punch biopsy revealed subcutaneous metastasis of ccRCC (Figure 2). The CT scan showed disease progression with new lesions on the lungs and in subcutaneous tissue. Due to his good condition, the multidisciplinary team decided to start immunotherapy with nivolumab 200 mg every two weeks as a third-line treatment. Unfortunately, he presented with disease progression six months later with new lung metastasis. At this time, the patient was ECOG status 1; therefore, it was decided to start axitinib 5 mg a day in fourth-line treatment, which was increased to 10 mg daily due to his good tolerance. Initially, he showed good clinical response with regression of subcutaneous lesions. However, two years after, subcutaneous lesions started to grow with new lesions at the thoracic wall (Figure 3). CT scan showed disease progression, with new subcutaneous lesions as well as a nodule in the pelvic cavity surrounding the spermatic cord and enlargement of lung lesions (Figure 4). After all these treatments, the patient showed ECOG status 1 without significant disease symptoms; hence, we decided to start fifth-line treatment with cabozantinib 60 mg daily.

The patient completed one and a half year of treatment with cabozantinib, with good tolerance; however, he again presented with disease progression in association with peritoneal carcinomatosis and ascites. At this time, it was decided to stop treatment with cabozantinib and give the patient the best supportive care.

## Discussion

We described a unique case report of a patient who is alive 15 years after being diagnosed with RCC, 13 of them with metastatic disease. Initially, the patient presented with local metastasis, and then progressed with pulmonary, cutaneous, and distant lymph nodes lesions, but with ECOG status 0/1. During this period, RCC treatment changed drastically with the development of new drugs; hence, the treatment sequence used in this clinical case was decided according to drug availability and the best knowledge about mRCC treatment. Besides multiple metastases, the patient had a good quality of life with good symptom control.

RCC is responsible for approximately 3% of adult tumors, and the five-year overall survival in the metastatic setting is only 12% [1,11]. In patients with a good-to-intermediate prognosis, the overall survival in a multicentric real-world study was 57 months (range: 51 to 70 months), and only 5% of the patients received five or more treatments lines [6]. Our patient is alive 156 months after mRCC diagnosis and completed five lines of treatment, which is very uncommon in the literature. To our knowledge, this is the first clinical case described in the literature with long-term survival who completed five lines of treatment, with overall survival of 156 months.

In the SUTENT clinical trial, which gave approval to sunitinib in the first-line setting, the median progression-free survival in patients with IMSDC favorable risk score was 14.1 (range: 13.4-17.1) months, and the median duration of treatment was six months. In this study, the overall survival was 26.4 months [11]. Our patient completed 60 months of sunitinib in first-line treatment of mRCC with stable disease, overall survival of 156 months which was extremely superior to what was found in this clinical trial.

In second-line treatment, clinical trials that approved everolimus (RANDON-1 and 4 clinical trials), the median progression-free survival was 4.9 (range: 4.0-5.5) months, and the probability of remaining progression-free 10 months after the start of treatment was 25% in the everolimus group [12]. In this clinical case, after more than 70 months with everolimus, the patient had stable disease, showing a good response to this treatment.

In third-line treatment, the patient completed six months of nivolumab due to disease progression, which was superior to that reported in the CheckMate 025 trial. In this trial, the use of nivolumab compared to everolimus improved the median overall survival by 5.4 months (25 months in the nivolumab arm versus 19.6 months in the everolimus arm) with the median progression-free survival of 4.6 months in the nivolumab treatment arm [13]. In the AXIS trial that compared second-line axitinib versus sorafenib, the median progression-free survival was 8.3 months in the first group and 5.7 months in the second group. No benefit in overall survival was seen in either treatment arm (20.1 months versus 19.2 months, respectively) [14]. The phase III trial METEOR compared cabozantinib to everolimus in tyrosine kinase inhibitor-refractory mRCC. The median progression-free survival was 13.8 months and the median overall survival was 21.4 months in the cabozantinib arm. This patient completed 24 months of axitinib and 18 months of cabozantinib, which was superior to what was seen in this clinical trial [15]. The patient is alive 156 months after the mRCC diagnosis, which is superior to what was seen in all clinical trials. Hence, our patient showed benefits in terms of overall survival that far outweighed that described in previous reports.

In the literature, the median disease treatment control after initiation of tyrosine kinase inhibitors in the first-line was 14 months, nine months for second-line treatment, seven months for third-line treatment, and six months for fourth-line. The imTOR achieved a median disease treatment control of eight and six months in second- and third-line use, respectively. It should be noted that the median overall survival after starting fourth-line treatment was 13 months [16]. Therefore, our patient achieved better disease control with this treatment sequence and better overall survival even after fourth-line treatment of 36 months.

A good risk score in the IMSDC risk score and the development of hypertension during treatment with tyrosine kinase inhibitor were associated with better overall survival in the literature [17,18]. Besides a good prognosis and IMSDC score, our patient developed hypertension during first- and second-line treatment with tyrosine kinase inhibitors which was associated with better overall survival.

RCC has the propensity for distance metastasis, but cutaneous metastasis is uncommon with an incidence of 2.8-6.8%. It mostly affects the scalp and neck and occurs during the first six months to five years after the initial diagnosis. Many large retrospective studies have reported the incidence of RCC with cutaneous metastasis and the different sites of cutaneous involvement, but no details of outcomes have been highlighted in these studies. Several single case studies highlighted the outcomes which were mostly poor, with overall survival of 10 to 17 months [4]. Our patient presented with cutaneous metastasis nine years after the initial diagnosis of metastatic ccRCC and the overall survival after this diagnosis was more than 48 months.

Nowadays, there is new evidence showing survival benefits with the use of immunotherapy in combination with tyrosine kinase inhibitors, especially in patients with poor-to-intermediate risk scores [8]. In patients with a good prognosis, the optimal sequence of treatment was not fully understood, but it appears that sequential administration of targeted therapy improves overall survival without cumulative toxicity [19]. In this case, the treatment sequence was made based on the approval of new drugs. Our patient presented an excellent response to everolimus with a progression-free survival of six years but had no response to immunotherapy with nivolumab. This situation might be explained by the fact that nivolumab was used in a later line and probably the tumor of our patient had some characteristics that predict a lack of response to immunotherapy. Indeed, programmed death-ligand 1 (PD-L1) expression was reported in 23% of ccRCC and can predict response to immunotherapy [20]. Unfortunately, PD-L1 expression was not measured in our patient.

As treatment options for mRCC are continuing to evolve, it is important that oncology centers share their experiences with various new drugs. In the era of personalized medicine in which biomarkers are becoming important, the study of each patient’s tumor individually may, in the near future, allow us to choose the best treatment sequence for each patient.

## Conclusions

In this clinical case, our patient is alive 156 months after metastatic ccRCC diagnosis, 48 of those with cutaneous metastasis without impacting his quality of life, which is uncommon. The treatment sequence used in this case with different tyrosine kinase inhibitors, mTOR inhibitors, and immunotherapy appears to be safe without cumulative toxicity and with good disease control. However, the best treatment sequence in this setting remains unknown. The landscape of metastatic ccRCC treatment is changing with the introduction of the combination of immunotherapy and tyrosine kinase inhibitors in the first line; however, for good prognosis, tyrosine kinase inhibitor monotherapy can be an option as first-line treatment, as shown in this clinical case.
